# Mediiert die schmerzspezifische Selbstwirksamkeit die Beziehung zwischen Depressivität und arbeitsbezogenen Faktoren bei nichtspezifischen chronischen Rückenschmerzen?

**DOI:** 10.1007/s00482-023-00701-0

**Published:** 2023-03-09

**Authors:** Petra Hampel, Anne Neumann

**Affiliations:** Institut für Gesundheits‑, Ernährungs- und Sportwissenschaften, Europa-Universität Flensburg, Auf dem Campus 1, 24943 Flensburg, Deutschland

**Keywords:** Mediationsanalyse, Chronischer Schmerz, Rehabilitation, Psychologische Faktoren, Depressionsskala, Mediation analysis, Chronic pain, Rehabilitation, Psychological factors, Depression scale

## Abstract

**Hintergrund:**

Die Chronifizierung nichtspezifischer Rückenschmerzen hängt vor allem mit psychologischen Faktoren zusammen. Wirkmechanismen psychologischer Faktoren wurden jedoch noch wenig bei nichtspezifischen chronischen Rückenschmerzen (CRS) untersucht, insbesondere nicht der Mediatoreffekt der schmerzspezifischen Selbstwirksamkeit.

**Fragestellung:**

Mediiert die schmerzspezifische Selbstwirksamkeit die langfristige Vorhersage arbeitsbezogener Faktoren durch die Depressivität?

**Methodik:**

Im Rahmen einer explorativen Sekundäranalyse wurden einfache Mediationsanalysen zur längsschnittlichen Vorhersage der subjektiven Erwerbsprognose sowie physischen und psychischen Arbeitsfähigkeit durch die Depressivität mit dem Mediator schmerzspezifische Selbstwirksamkeit bei 382 Personen mit nichtspezifischen CRS in der stationären Rehabilitation durchgeführt.

**Ergebnisse:**

Die Befunde sprechen dafür, dass die Depressivität zu Rehabilitationsbeginn die Ausprägungen in allen drei arbeitsbezogenen Kennwerten 24 Monate nach der Rehabilitation vorhersagte und die schmerzspezifische Selbstwirksamkeit 12 Monate nach der Rehabilitation diesen Zusammenhang vermittelte.

**Schlussfolgerung:**

Bei der Behandlung nichtspezifischer CRS sollte zur nachhaltigen Verbesserung des arbeitsbezogenen Rehabilitationserfolgs insbesondere die schmerzspezifische Selbstwirksamkeit, aber auch die Depressivität berücksichtigt werden.

**Zusatzmaterial online:**

Die Online-Version dieses Beitrags (10.1007/s00482-023-00701-0) enthält weitere Tabellen.

## Abstract (en)

**Background:**

The transition from acute to non-specific chronic low back pain (CLBP) is especially associated with psychological factors. However, working mechanisms of psychological factors have been little examined in non-specific CLBP, especially the mediator effect of pain self-efficacy.

**Objective:**

Does pain self-efficacy mediate the long-term prediction of work-related factors by depressive symptoms?

**Methods:**

Within the framework of an exploratory secondary analysis, simple mediation analyses were conducted to longitudinally predict prognosis of gainful employment, as well as subjective physical and mental work ability by depressive symptoms mediated by pain self-efficacy in 382 inpatients with non-specific CLBP.

**Results:**

The findings suggest that depressive symptoms prior to rehabilitation predicted levels of all three work-related factors 24 months after rehabilitation, and pain self-efficacy 12 months after rehabilitation mediated this relationship.

**Conclusion:**

To improve the success of work-related rehabilitation in the long-term, pain self-efficacy in particular, but also depressive symptoms should be targeted by treatments of non-specific CLBP.

Psychologische Faktoren üben einen entscheidenden Einfluss auf die Chronifizierung von Rückenschmerzen aus. Insbesondere depressive Symptome gehen mit nichtspezifischen chronischen Rückenschmerzen einher und stehen im Zusammenhang mit einem erhöhten Risiko von Frühberentungen. Als vermittelnder Schutzfaktor hat sich in ersten Studien die schmerzspezifische Selbstwirksamkeit erwiesen. In diesem Beitrag wird die schmerzspezifische Selbstwirksamkeit als Mediator in der Beziehung zwischen Depressivität und arbeitsbezogenen Faktoren untersucht.

## Hintergrund und Fragestellung

Chronische Rückenschmerzen (CRS) sind durch eine Dauer von mindestens 3 Monaten gekennzeichnet und gehen häufig mit psychischen Komorbiditäten wie Depressionen und Angststörungen einher, die das Risiko von Frühberentungen erhöhen [23]. Insgesamt entfielen im Jahr 2021 5,25 % aller stationären Leistungen zur medizinischen Rehabilitation und sonstigen Leistungen zur Teilhabe am Arbeitsleben auf die nichtspezifischen CRS [7]. Nachhaltige Rehabilitationseffekte waren jedoch bislang klein und gingen selten über eine 12-Monats-Katamnese (MK) hinaus [28]. Somit besteht auch heute noch ein dringender Handlungsbedarf hinsichtlich der Sekundärprävention von CRS, die durch eine frühzeitige Identifikation von Risikofaktoren und Zuweisung zu bedarfsgerechten Interventionen geleistet werden kann [16].

In Modellen der Schmerzchronifizierung wird von einem biopsychosozialen Krankheitsgeschehen ausgegangen, wobei psychologische Faktoren eine entscheidende Rolle spielen. So sollen im Rahmen des Fear-avoidance-Modells und des erweiterten Avoidance-endurance-Modells ungünstige Schmerzkognitionen und Emotionen wie Furcht, Angst und Depression den Chronifizierungsprozess vermitteln (vgl. [12]). Empirische Befunde konnten den ungünstigen Einfluss von Depressivität und maladaptiven Kognitionen auf die Entwicklung von CRS bestätigen [18]. So untermauerten frühere Studien in der orthopädischen Rehabilitation den Zusammenhang: Die Depressivität mediierte die ungünstigen Effekte des Vermeidungsverhaltens zu Rehabilitationsbeginn auf die körperliche und psychische Beeinträchtigung 12 Monate nach der Rehabilitation [24] bzw. die ungünstigen Effekte der Angst-Vermeidungs-Kognitionen zu Rehabilitationsbeginn auf die körperliche Lebensqualität 24 Monate nach der Rehabilitation [22]. Insgesamt liegen noch eher wenige Studien zu den langfristigen Wirkmechanismen psychologischer Faktoren vor [27], die mithilfe von Mediationsanalysen gut erforscht werden können.

Neuere Studien rückten zudem die Bedeutung der schmerzspezifischen Selbstwirksamkeit als psychologischer Schutzfaktor in den Vordergrund. Selbstwirksamkeit wird definiert als die subjektive Gewissheit, neue oder schwierige Anforderungssituationen aufgrund eigener Kompetenz bewältigen zu können [2]. Eine hohe Selbstwirksamkeitserwartung steht im Zusammenhang mit einer Intentionsbildung sowie Planung, Durchführung und Aufrechterhaltung gesundheitsförderlichen Verhaltens und könnte die beeinträchtigenden Effekte der Depressivität auf die Schmerzchronifizierung vermitteln. Eine Stärkung der schmerzspezifischen Selbstwirksamkeit durch Selbstmanagementtechniken im Rahmen eines biopsychosozialen Schmerzmanagementprogramms zeigte sich in einer aktuelleren Studie als stärkster Prädiktor für eine kurzfristige Verbesserung des Funktionsstatus bei CRS [27].

Durch die Berücksichtigung der schmerzspezifischen Selbstwirksamkeit könnten bislang widersprüchliche Befunde zum Einfluss der Depressivität auf schmerzbezogene Kennwerte bei nichtspezifischen CRS besser erklärt werden. So sprechen einige Studien für den negativen Zusammenhang der Depressivität mit der schmerzspezifischen Selbstwirksamkeit [17, 26]. In weiteren Studien hing die schmerzspezifische Selbstwirksamkeit ihrerseits mit der wahrgenommenen Beeinträchtigung durch die Schmerzen zusammen [17, 21].

Der Einfluss der schmerzspezifischen Selbstwirksamkeit auf arbeitsbezogene Kennwerte ist wenig erforscht

Des Weiteren wird zunehmend die Bedeutung arbeitsbezogener Risikofaktoren diskutiert (vgl. [1]), wobei jedoch der Einfluss der schmerzspezifischen Selbstwirksamkeit auf arbeitsbezogene Kennwerte wenig erforscht ist. In einer Studie war die ungünstige Selbstwirksamkeit der stärkste Prädiktor für eine langfristige Arbeitsunfähigkeit bei nichtspezifischen muskuloskeletalen Erkrankungen [3].

Ein vermittelnder Einfluss der schmerzspezifischen Selbstwirksamkeit wurde vor allem in zwei Studien bei CRS nahegelegt: In einem 1‑Jahres-Längsschnitt vermittelten nicht die Angst-Vermeidungs-Kognitionen, sondern die schmerzspezifische Selbstwirksamkeit den ungünstigen Einfluss der Schmerzintensität auf die Schmerzbeeinträchtigung [5]. Für die vorliegende Arbeit ist jedoch folgender Befund sehr relevant: Die schmerzspezifische Selbstwirksamkeit mediierte die Beziehung zwischen hoher Depressivität zu Rehabilitationsbeginn und der erhöhten Schmerzintensität zum Rehabilitationsende [26].

Um den bedeutenden Einfluss komorbider psychischer Beeinträchtigungen in der Rehabilitation orthopädischer Erkrankungen zu berücksichtigen, wurde 2013 die verhaltensmedizinisch orthopädische Rehabilitation (VMO) etabliert [6]. Längsschnittliche Wirkmechanismen der psychologischen Risikofaktoren in der Schmerzchronifizierung wurden jedoch bislang für die VMO kaum berichtet. Moderatoreffekte der Depressivität auf die schmerzspezifische Selbstwirksamkeit wurden in unserer Analyse der 12-MK nahegelegt [11]. Studien zu langfristigen Mediatoreffekten der schmerzspezifischen Selbstwirksamkeit liegen bislang nicht vor. Dementsprechend war das Ziel der vorliegenden explorativen Sekundäranalyse einer Interventionsstudie, den vermittelnden Einfluss der schmerzspezifischen Selbstwirksamkeit auf die langfristige Vorhersage von arbeitsbezogenen Kennwerten durch die Depressivität in der VMO zu untersuchen. So wurde geprüft, ob die ungünstigen Effekte der Depressivität zu Rehabilitationsbeginn auf die subjektive Gefährdung der Erwerbsprognose sowie die physische und psychische Arbeitsfähigkeit zur 24-MK durch die schmerzspezifische Selbstwirksamkeit zur 12-MK vermittelt werden. Somit sollte der erste Befund von Skidmore et al. [26] sowohl auf die langfristige Vorhersage als auch auf die arbeitsbezogenen Kennwerte erweitert werden.

## Material und Methoden

### Studiendesign und Untersuchungsmethode

Zur Beantwortung der Fragestellung wurden längsschnittliche einfache Mediationsanalysen durchgeführt, die auf einer explorativen Sekundäranalyse des Datensatzes des Forschungsprojekts Debora zur Wirksamkeit des kognitiv-behavioralen kombinierten Schmerzkompetenz- und Depressionspräventionstrainings in der stationären VMO basierten (vgl. [11]). Im Rahmen dieser kontrollierten Multicenterstudie erhielt die Kontrollgruppe (KG) 4 Einheiten eines Schmerzbewältigungstrainings und die Interventionsgruppe (IG) das kombinierte Training zur Schmerzkompetenz und Depressionsprävention mit 8 Einheiten (vertiefend siehe [20]). Um den unterschiedlichen zeitlichen Umfang zu kompensieren, nahm die KG zusätzlich noch an Entspannungskursen teil. In diese Auswertungen gingen Daten der Zeitpunkte zu Rehabilitationsbeginn (t_0_) sowie 12 Monate (t_3_) und 24 Monate nach der Rehabilitation (t_4_) ein. Die Datenerhebung fand zu Rehabilitationsbeginn mittels schriftlicher Fragebögen und im ärztlichen Aufnahmegespräch statt. Die Fragebogenerhebung zur 12- und 24-MK erfolgte postalisch von Oktober 2016 bis Dezember 2017.

Die Studie wurde von der Ethikkommission der Deutschen Gesellschaft für Psychologie genehmigt. Alle Teilnehmenden unterschrieben schriftliche Erklärungen zur informierten Einwilligung.

### Ein- und Ausschlusskriterien

In die Studie wurden Personen mit nichtspezifischen CRS eingeschlossen, die seit mindestens 6 Monaten CRS, eine Hauptdiagnose nach Internationaler statistischer Klassifikation der Krankheiten und verwandter Gesundheitsprobleme (ICD-10) in den Bereichen M51, M53 oder M54 und ausreichende deutsche Sprachkenntnisse aufwiesen. Als Ausschlusskriterien galten schwere psychiatrische oder somatische Komorbiditäten, Unfälle/Operationen in den letzten 6 Monaten und eine bestehende Schwangerschaft.

### Stichprobe

Die 382 Teilnehmenden waren zu Rehabilitationsbeginn im Durchschnitt 53,55 Jahre alt (Standardabweichung [*SD*] = 5,79 Jahre; Altersbereich: 32–64 Jahre) und zu 81,2 % weiblich. Die mittlere Schmerzdauer betrug 15,85 Jahre (*SD* = 11,24 Jahre; Tab. 1) und 50,3 % der Teilnehmenden zeigten klinische Auffälligkeiten auf der Allgemeinen Depressionsskala (ADS; [14]). Mit 51,8 % waren etwas mehr als die Hälfte der Teilnehmenden im Chronifizierungsstadium II [10]. Die Teilnehmenden der beiden experimentellen Gruppen unterschieden sich in den aufgeführten Kennwerten nicht signifikant voneinander.

**Tab. 1:** Stichprobencharakteristik

Kennwert	*N*^*a*^	
**Soziodemografische Daten**		
*Alter, Jahre, M* *±* *SD*	382	53,55 ± 5,79
*Geschlecht, weiblich, n (%)*	382	310 (81,2 %)
*Familienstand, verheiratet, n (%)*	382	248 (64,9 %)
*Schulbildung, n (%)*	378	–
Niedrig	–	73 (19,3 %)
Mittel	–	194 (51,3 %)
Hoch	–	111 (29,4 %)
**Arbeitsbezogene Daten**		
*Erwerbstätig, n (%)*	365	334 (91,5 %)
*Arbeitsunfähigkeitstage (in den letzten 3 Monaten), mehr als 2 Wochen, n (%)*	382	186 (48,7 %)
*Subjektive Erwerbsprognose, M* *±* *SD*	382	1,24 ± 1,08
*Subjektive physische Arbeitsfähigkeit, M* *±* *SD*	382	2,81 ± 0,97
*Subjektive psychische Arbeitsfähigkeit, M* *±* *SD*	382	2,82 ± 0,96
**Schmerzbezogene Daten**		
*Schmerzdauer, Jahre, M* *±* *SD*	342	15,85 ± 11,24
*Durchschnittliche Schmerzintensität, M* *±* *SD*	372	4,89 ± 1,87
*Chronifizierungsstadium, MPSS*^*b*^*, n (%)*	382	–
I	–	95 (24,9 %)
II	–	198 (51,8 %)
III	–	89 (23,3 %)
**Psychologische Daten**		
*Depressivität, ADS*^*c*^* ≥* *23, n (%)*	382	192 (50,3 %)
*Schmerzspezifische Selbstwirksamkeit, M* *±* *SD*	382	39,05 ± 11,61

*M* Mittelwert, *SD* „standard deviation“ (Standardabweichung)

^a^Unterschiedliche Größen für *N* ergaben sich aufgrund fehlender Werte in den Fragebögen

^b^*MPSS* Mainz Pain Staging System (Mainzer Stadienmodell der Schmerzchronifizierung [10])

^c^*ADS* Allgemeine Depressionsskala [14]

Von den 2075 angesprochenen Personen nahmen 1306 an der Studie teil (Ausschöpfungsrate: 62,94 %). Während der Rehabilitation brachen 100 Personen die Studienteilnahme ab (Drop-out-Rate: 7,66 %) und bis zur 24-MK insgesamt 833 Personen (Drop-out-Rate insgesamt: 63,78 %). Schließlich wurden 91 Teilnehmende aufgrund umfangreich fehlender Daten ausgeschlossen (6,97 %). In der Drop-out-Analyse unterschieden sich Personen, die die Studie abbrachen, in der KG und IG nicht signifikant; im Vergleich zu den Studienteilnehmenden waren Personen, die die Studie abbrachen, aber insgesamt jünger, eher männlich, seltener verheiratet und seltener mindestens halbtags berufstätig. Ferner hatten sie eine höhere Schmerzdauer und durchschnittliche Schmerzintensität. In die Intention-to-treat(ITT)-Analyse gingen insgesamt Daten von 1225 Personen ein.

### Messinstrumente

Zwei psychologische Kennwerte wurden herangezogen: Anhand der ADS wurde die *Depressivität* bestimmt [14]; hierbei wurde der Summenwert von 20 Items mit einer Antwortskalierung von 0 (selten oder überhaupt nicht) bis 3 (meistens, die ganze Zeit) berechnet. Die schmerzbezogene Selbstwirksamkeit wurde mit dem Fragebogen zur Erfassung der *schmerzspezifischen Selbstwirksamkeit* (FESS) in seiner deutschen Version ermittelt [17]. Die 10 Items müssen mit einer Antwortskalierung von 1 (gar nicht überzeugt) bis 6 (vollkommen überzeugt) eingeschätzt werden.

Im Bereich der arbeitsbezogenen Kennwerte wurde die *subjektive Gefährdung der Erwerbsprognose* anhand der Skala zur Messung der subjektiven Prognose der Erwerbstätigkeit (SPE) erfasst, die über Einzelitems die derzeitige Berufstätigkeit, Erwerbsfähigkeit und das Rentenbegehren erfragt [19]. Ferner wurden 2 Einzelitems des Gesamtindex der Arbeitsfähigkeit (Work Ability Index [WAI]) herangezogen, die die *subjektive physische und psychische Arbeitsfähigkeit* abbilden [13].

Das *Chronifizierungsstadium* wurde mithilfe des Mainzer Stadienmodells der Schmerzchronifizierung (Mainz Pain Staging System [MPSS]) bestimmt [10], das im ärztlichen Vorgespräch ermittelt wurde.

### Statistische Auswertung

**Abb. 1:**
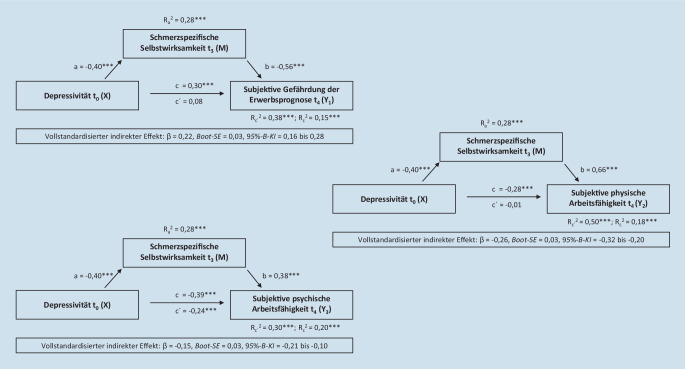
Pfadmodelle der drei Fragestellungen mit standardisierten Regressionskoeffizienten und dem Anteil aufgeklärter Varianz (korrigiertes *R*^*2*^) der Mediationsanalyse bei *N* = 382. *t*_*0*_ Rehabilitationsbeginn, *t*_*3*_ 12 Monate nach der Rehabilitation, *t*_*4*_ 24 Monate nach der Rehabilitation. *95* *%-B-KI* biaskorrigiertes 95 %-Bootstrap-Konfidenzintervall, *Boot-SE* „bootstrap standard error“ (Bootstrap-Standardfehler). ***p < 0,001

Die Mediationsanalysen wurden mit IBM SPSS Statistics 26 und dem Befehl PROCESS [15] durchgeführt. Mediationsanalysen werden als Pfadanalysen formalisiert und stellen eine Erweiterung multipler Regressionsanalysen dar [8]. Hierdurch lassen sich relativ komplexe Zusammenhangsstrukturen untersuchen. In einem pfadanalytischen Modell kann eine Variable sowohl unabhängige als auch abhängige Variable sein. So ist im vorliegenden Fall die vermittelnde Variable „schmerzspezifische Selbstwirksamkeit“, die zwischen der Depressivität als unabhängige Variable (X) und den arbeitsbezogenen Kennwerten als abhängige Variablen (Y_1–3_) vermitteln soll, sowohl eine unabhängige als auch eine abhängige Variable. Vermittelnde Variablen werden auch Mediatorvariablen genannt. Es werden in einer einfachen Mediationsanalyse 3 direkte Einflüsse (Pfade/Effekte), ein indirekter Pfad und ein totaler Effekt bestimmt: Im vorliegenden Fall: a = direkter Pfad von Depressivität zu Rehabilitationsbeginn (X) auf die schmerzspezifische Selbstwirksamkeit zur 12-MK (M), b = direkter Pfad der schmerzspezifischen Selbstwirksamkeit zur 12-MK (M) auf die subjektive Gefährdung der Erwerbsprognose (Y_1_, im Modell 1), die subjektive physische Arbeitsfähigkeit (Y_2_, im Modell 2) bzw. die subjektive psychische Arbeitsfähigkeit (Y_3_, im Modell 3) zur 24-MK und c′ = direkter Pfad von Depressivität zu Rehabilitationsbeginn (X) auf die subjektive Gefährdung der Erwerbsprognose (Y_1_, im Modell 1), die subjektive physische Arbeitsfähigkeit (Y_2_, im Modell 2) bzw. die subjektive psychische Arbeitsfähigkeit zur 24-MK (Y_3_, im Modell 3); „ohne Mediator“. Der indirekte Effekt (ab) ist der Effekt, den eine unabhängige Variable (hier Depressivität) vermittelt über eine andere Variable (hier schmerzspezifische Selbstwirksamkeit) auf eine abhängige Variable ausübt (hier arbeitsbezogene Kennwerte). Hierbei geht das Produkt der beiden Pfadkoeffizienten der direkten Pfade a und b ein. Der totale Effekt ist der Gesamteffekt, den die unabhängige Variable (hier Depressivität) auf die abhängige Variable (hier arbeitsbezogene Kennwerte) hat und der Pfad c in Abb. 1 entspricht. Dies ist die Addition des indirekten Effekts (ab) und des direkten Effekts von c′ („mit dem Mediator“).

Insbesondere wird überprüft, ob sich unter Hereinnahme des Mediators (direkter Effekt c′) der Gesamteffekt c abschwächt oder verschwindet („Ist der direkte Effekt niedriger als der totale Effekt?“) und ob signifikante indirekte Effekte vorliegen, die die Mediationseffekte statistisch absichern (vertiefend siehe [8, 9, 15]). Die Fragestellung der vorliegenden Analyse zielte auf den indirekten Effekt für die 3 Modelle ab.

Pearson-Produkt-Moment-Korrelationen untermauerten, dass Alter und Geschlecht keine Bezüge zu den Mediationsvariablen hatten (*p* > 0,01) und daher nicht in die Mediationsanalyse eingeschlossen werden mussten. Es wurden jedoch das Chronifizierungsstadium und die Zuordnung zur KG/IG als Kovariaten in die 3 Modelle aufgenommen, um mögliche Einflüsse zu kontrollieren. Die Befunde der Per-protocol-Analyse (pp) mit *N* = 382 wurden durch Analysen nach multipler Imputation (MI) mit 10 Iterationen an *N* = 1225 validiert. Aufgrund des explorativen Charakters wurde ein Signifikanzniveau von 5 % festgelegt.

Bis auf die Normalverteilungsannahme waren alle Voraussetzungen für Mediationsanalysen gegeben; jedoch kann diese Verletzung aufgrund der Verwendung der Bootstrap-Konfidenzintervalle und einer umfangreichen Stichprobe vernachlässigt werden [9, 15]. Die Ergebnisse der Mediationsanalysen wurden anhand des korrigierten *R*^*2*^ interpretiert, wobei die Effektstärke *R*^*2*^ als klein (0,02), moderat (0,13) und groß (0,26) bewertet wurde [4]. Die statistische Absicherung erfolgte mithilfe von Bootstrap-Konfidenzintervallen des indirekten Effekts (Anzahl der Bootstrap-Stichproben: 5000). Hierbei wurden die Bootstrap-Konfidenzintervalle der nicht-, teil- und vollstandardisierten Effektgrößen betrachtet [9, 15]; umschließt das Intervall zwischen der unteren und oberen Grenze des Bootstrap-Konfidenzintervalls aller drei Effektgrößen nicht die Null, kann ein bedeutsamer Mediationseffekt angenommen werden. Es wurde laut Hayes [15] nicht zwischen partieller und vollständiger Mediation unterschieden.

## Ergebnisse

Die partiellen Korrelationen der Modellvariablen ergaben statistisch signifikante Zusammenhänge (Tab. 2).

**Tab. 2:** Deskriptive Statistik und partielle Korrelationen der Modellvariablen unter Kontrolle der Variablen Kontroll‑/Interventionsgruppe und Chronifizierungsstadium (MPSS^a^; [10]) für *N* = 382

Kennwert	*M* ± *SD*	ADS^b^	FESS^c^	SPE^d^	Phy^e^	Psy^f^
Depressivität t_0_	23,02 ± 11,44	1,00	–	–	–	–
Selbstwirksamkeit t_3_	42,52 ± 12,97	−0,42	1,00	–	–	–
Erwerbsprognose t_4_	1,21 ± 1,18	0,31	−0,58	1,00	–	–
Physische Arbeitsfähigkeit t_4_	3,07 ± 1,09	−0,29	0,66	−0,63	1,00	–
Psychische Arbeitsfähigkeit t_4_	3,14 ± 1,16	−0,39	0,47	−0,53	0,58	1,00

*M* Mittelwert, *SD* „standard deviation“ (Standardabweichung), *t*_*0*_ Rehabilitationsbeginn, *t*_*3*_ 12 Monate nach der Rehabilitation, *t*_*4*_ 24 Monate nach der Rehabilitation

^a^*MPSS* Mainz Pain Staging System (Mainzer Stadienmodell der Schmerzchronifizierung [10])

^b^*ADS* Allgemeine Depressionsskala [14]

^c^*FESS* Fragebogen zur Erfassung der schmerzspezifischen Selbstwirksamkeit [15]

^d^*SPE* Skala zur Messung der subjektiven Prognose der Erwerbsfähigkeit [17]

^e^*Phy* Subjektive physische Arbeitsfähigkeit

^f^*Psy* Subjektive psychische Arbeitsfähigkeit [11]

*p* < 0,001

Insgesamt konnten somit alle drei einfachen Mediationsanalysen berechnet werden, in die die Depressivität zu Rehabilitationsbeginn als unabhängige Variable (X) und die schmerzspezifische Selbstwirksamkeit zur 12-MK als Mediatorvariable (M) eingingen. Als abhängige Variable (Y) wurde jeweils die subjektive Gefährdung der Erwerbsprognose (Modell 1), die subjektive physische Arbeitsfähigkeit (Modell 2) bzw. die subjektive psychische Arbeitsfähigkeit (Modell 3) zur 24-MK herangezogen. Für die vier Pfade (a, b, c, c′) und den indirekten Effekt (ab) werden die standardisierten Koeffizienten (β) berichtet.

In allen Modellen stellten sich Mediationseffekte dar (vgl. Tabellen im Online-Zusatzmaterial). So umfassten die biaskorrigierten 95 %-Bootstrap-Konfidenzintervalle (95 %-B-KI) in den nicht-, teil- und vollstandardisierten Effektgrößen nicht den Wert Null. Die Effektstärken aller totalen Effekte (c) waren moderat; dagegen waren die Effektstärken der direkten Effekte (c′), wenn die schmerzspezifische Selbstwirksamkeit als Mediator hereingenommen wurde, groß. Schließlich wurden alle Mediationseffekte, die sich in den pp-Analysen ergeben hatten, durch die MI-Analysen bestätigt (vollstandardisierter indirekter Effekt: Modell 1: β = 0,11; Bootstrap-Standardfehler [Boot-SE] = 0,01, 95 %-B-KI = 0,09 bis 0,14; Modell 2: β = −0,12; Boot-SE = 0,01, 95 %-B-KI = −0,15 bis −0,10; Modell 3: β = −0,08; Boot-SE = 0,01, 95 %-B-KI = −0,11 bis −0,06).

### Mediationsanalyse

#### Mediationsmodell 1.

Das Pfadmodell der Mediationsanalyse in Abb. 1 verdeutlicht, dass die Varianzaufklärung der subjektiven Gefährdung der Erwerbsprognose (Y_1_) durch die Hereinnahme der schmerzspezifischen Selbstwirksamkeit von 15 % auf 38 % stieg. Der totale Effekt der Depressivität auf die subjektive Gefährdung der Erwerbsprognose wies als direkter Effekt bei Berücksichtigung der schmerzspezifischen Selbstwirksamkeit im Mediationsmodell keine Signifikanz mehr auf. Somit sagte ein hoher Depressivitätswert eine geringe schmerzspezifische Selbstwirksamkeit vorher und eine geringe schmerzspezifische Selbstwirksamkeit sagte in Folge eine hohe subjektive Gefährdung der Erwerbsprognose vorher.

#### Mediationsmodell 2.

Gleichgerichtete Befunde ergaben sich für das zweite Pfadmodell: Der Anteil der erklärten Varianz für die subjektive physische Arbeitsfähigkeit (Y_2_) erhöhte sich von 18 % auf 50 %, wenn die schmerzspezifische Selbstwirksamkeit als Mediator im Zusammenhang zwischen dem Depressivitätswert und der subjektiven physischen Arbeitsfähigkeit berücksichtigt wurde (Abb. 1). Auch hier war der totale Effekt der Depressivität auf die subjektive physische Arbeitsfähigkeit als direkter Effekt bei Hereinnahme der schmerzspezifischen Selbstwirksamkeit im Mediationsmodell nicht mehr signifikant.

#### Mediationsmodell 3.

Die Varianzaufklärung für das Zielkriterium „subjektive psychische Arbeitsfähigkeit“ (Y_3_) stieg von 20 % auf 30 %, wenn die schmerzspezifische Selbstwirksamkeit als Mediator in das Modell hereingenommen wurde (Abb. 1). Der direkte Effekt verringerte sich zwar, wenn die schmerzspezifische Selbstwirksamkeit als Mediator berücksichtigt wurde, blieb aber signifikant.

## Diskussion

Die vorliegende Analyse zu den längsschnittlichen Wirkzusammenhängen im Chronifizierungsprozess bei Personen in der VMO sprechen dafür, dass

- eine hohe Depressivität eine geringere schmerzspezifische Selbstwirksamkeit vorhersagt,
- diese wiederum ungünstige Ausprägungen in den arbeitsbezogenen Kennwerten vorhersagt und schließlich
- die schmerzspezifische Selbstwirksamkeit 12 Monate nach Ende der Rehabilitation die Vorhersage der arbeitsbezogenen Kennwerte zur 24-MK durch die Depressivität zu Rehabilitationsbeginn mediiert.

Demnach konnten erste Befunde zu den Wirkmechanismen bei nichtspezifischen CRS in der Literatur bestätigt bzw. erweitert werden: Bereits frühere Studien belegten den Zusammenhang zwischen Depressivität und schmerzspezifischer Selbstwirksamkeit im Querschnitt [17] wie auch im Längsschnitt mit kurzem Zeitintervall [26]. Auch in unserer früheren Analyse besaß die Depressivität zu Rehabilitationsbeginn einen Moderatoreffekt auf die schmerzspezifische Selbstwirksamkeit im 1‑Jahres-Längsschnitt [11]. Außerdem stimmen die vorliegenden Ergebnisse mit gleichgerichteten Befunden zum ungünstigen Effekt der geringen Selbstwirksamkeit auf die langfristige Arbeitsunfähigkeit bei muskuloskeletalen Erkrankungen überein [3]. Schließlich lagen bislang nur zwei Befunde zu den Mediationseffekten der schmerzspezifischen Selbstwirksamkeit vor, wobei in einer Studie der vermittelnde Effekt auf die Vorhersage der Schmerzbeeinträchtigung durch die Schmerzintensität im 1‑Jahres-Verlauf untersucht wurde [5]. Von besonderer Relevanz für das Ziel und die Fragestellung ist jedoch, dass die Mediationseffekte der schmerzspezifischen Selbstwirksamkeit auf die Beziehung zwischen der Depressivität zu Rehabilitationsbeginn und der Schmerzintensität zur Posterhebung einer 4‑wöchigen Rehabilitationsmaßnahme bei Skidmore et al. [26] sowohl auf die arbeitsbezogenen Kennwerte als auch auf den längeren Beobachtungszeitraum von 2 Jahren erweitert werden konnten.

Der Zusammenhang zwischen Depressivität und schmerzspezifischer Selbstwirksamkeit wurde bestätigt

Implikationen der vorliegenden Ergebnisse beziehen sich einerseits auf den längsschnittlichen Zusammenhang der schmerzspezifischen Selbstwirksamkeit mit den arbeitsbezogenen Kennwerten. Hier spiegelt sich die große Bedeutung des erfahrungsgeleiteten Lernens und des Alltagstransfers der in der Rehabilitation erlernten Handlungsweisen wider. Durch eine noch bessere Verzahnung von bewegungs- und psychotherapeutischen Maßnahmen im interprofessionellen Team [25] und die langfristige Förderung der Selbstwirksamkeit im Alltag und Arbeitsleben im Rahmen von Nachsorgemaßnahmen ließen sich die bisherigen kurzfristigen Rehabilitationserfolge verstetigen.

Andererseits legt die Bedeutung der schmerzspezifischen Selbstwirksamkeit für die langfristige Vorhersagekraft der Depressivität nahe, dass die schmerzspezifische Selbstwirksamkeit den Schlüssel zur Veränderung sich überlappender Prozesse darstellen könnte, die dann als Schutzfaktor auf die ungünstige Beziehung zwischen Depressivität und arbeitsbezogenen Kennwerten einwirkt (vgl. [22, 24]). Demnach wird angedeutet, im Sinne der Fear-avoidance- bzw. Avoidance-endurance-Modelle die funktionalen kognitiven Schmerzverarbeitungsstrategien zu stärken. Eine aktuellere Studie spricht jedoch dafür, insbesondere die schmerzspezifischen Selbstwirksamkeitserwartungen zu fördern: Die schmerzspezifische Selbstwirksamkeit war der „Treiber“ der kurzfristigen Verbesserung des Funktionsstatus bei CRS, wobei ihre Vorhersagekraft 6‑ bis 10-fach höher war als die der Katastrophisierung und Bewegungsangst [27].

### Limitationen

Obwohl die Befunde frühere Ergebnisse bestätigen bzw. erweitern konnten, lässt sich kritisch anmerken, dass zu Beginn der Erforschung solcher längsschnittlicher Wirkzusammenhänge im Modell zunächst nur ein Mediator eingeschlossen wurde. Somit müssen zukünftig komplexere Modelle überprüft werden. Allerdings deuten die hohen Varianzaufklärungen insbesondere für die subjektive Gefährdung der Erwerbsprognose und die subjektive physische Arbeitsfähigkeit darauf hin, dass eher keine anderen Drittvariablen zu berücksichtigen sind. Zudem wurde die längsschnittliche Vorhersage der Depressivität auf die arbeitsbezogenen Kennwerte untersucht, wobei zwar die Effektrichtung interpretiert werden kann, in der Literatur jedoch auch Evidenz für eine bidirektionale Wirkrichtung der Depressivität im Chronifizierungsprozess besteht. Des Weiteren sind kausale Aussagen nicht möglich, weil die Prädiktoren nicht experimentell manipuliert wurden. Schließlich ist aufgrund der Art und Ziehung der Stichprobe die Generalisierbarkeit der Ergebnisse eingeschränkt. So wurden die Teilnehmenden in der stationären VMO gewonnen; sie waren erwartungsgemäß mehrheitlich weiblich, wiesen mit im Mittel knapp 16 Jahren eine lange Schmerzdauer auf, befanden sich zur Hälfte im Chronifizierungsstadium II und zu einem Viertel im Stadium III und hatten zur Hälfte eine klinisch auffällige Depressivität, sodass eine häufige psychische Komorbidität angenommen werden kann. Allerdings weist die Studie auch Stärken auf. So wurden die Wirkmechanismen im 2‑Jahres-Verlauf untersucht, die pp-Befunde durch MI-Analysen bestätigt und die Effekte durch die konfundierende Variable „Chronifizierungsstadium“ bereinigt.

## Fazit für die Praxis

- Die Mediationsbefunde sprechen dafür, dass im Rahmen der Rehabilitation bei CRS ein Aufbau der schmerzspezifischen Selbstwirksamkeit erfolgen sollte.
- Dies könnte im Rahmen der stationären Rehabilitation erfolgen, indem insbesondere bewegungs- und psychotherapeutische Maßnahmen noch besser verzahnt werden.
- Ferner scheint für die nachhaltige Verbesserung arbeitsbezogener Kennwerte eine Implementierung psychotherapeutischer Behandlungselemente im Rahmen von Nachsorgemaßnahmen nötig, die auf den Arbeitskontext angewendet werden.
- Schließlich wurde nochmals die Bedeutung der Depressivität für den Chronifizierungsprozess deutlich, sodass Komponenten von Emotionsregulationstrainings und störungsspezifische Elemente zur Prävention psychischer Komorbiditäten noch mehr Berücksichtigung in der Rehabilitation bei nichtspezifischen CRS finden sollten.

## Supplementary Information

Mediationen 1–3
