# Supplementary material for: Is the relationship between depressive symptoms and work-related factors mediated by pain self-efficacy in non-specific chronic low back pain?
Source: Schmerz. 2023 Mar 9;38(5):335–42. [Article in German] doi: 10.1007/s00482-023-00701-0 (PMC11420306; doi:10.1007/s00482-023-00701-0)
Supplement: Supplementary file 1 — Mediationen 1–3 [file 482_2023_701_MOESM1_ESM.pdf]

Mediation 1: Zusammenhang zwischen Depressivität zu Rehabilitationsbeginn und subjektiver Gefährdung der Erwerbsprognose zur 24-Monatskatamnese mit schmerzspezifischer Selbstwirksamkeit (FESS) zur 12-Monatskatamnese als Mediator sowie MPSS und experimentelle Bedingung (Kontrollgruppe vs. Interventionsgruppe) als Kovariaten (*N*=382)

		**Kriterium**						
		***M* (Schmerzspezifische Selbstwirksamkeit)**				***Y* (Subjektive Gefährdung der Erwerbsprognose)**		
**Prädiktor**		*Koeff.*	*SE*	*p*		*Koeff.*	*SE*	*p*
X (Depressivität)					*c*	0,30	0,05	<,001
X (Depressivität)	*a*	-0,40	0,04	<,001	*c´*	0,08	0,05	,092
M (FESS)		-	-	-	*b*	-0,56	0,05	<,001
C_1 (MPSS) [1]	f_1	-0,36	0,06	<,001	g_1	0,08	0,06	,208
C_2 (BED) [1]	f_2	0,18	0,09	,046	g_2	0,07	0,08	,933
		$R_a^2 = 0,28$, $F(3, 378) = 47,88$, $p < ,001$				$R_{c'}^2 = 0,38$, $F(4, 377) = 58,22$, $p < ,001$		

Vollstandardisierter indirekter Effekt (*ab*)	β	Boot SE	95%-B-KI
	0,22	0,03	0,16 - 0,28

Anmerkungen: MPSS = Mainzer Stadienmodell der Schmerzchronifizierung, BED = experimentelle Bedingung; Koeff.: β-Koeffizient, *SE*: Standardfehler, *p*: Signifikanzwert, Boot *SE*: Bootstrap-Standardfehler, 95%-B-KI: 95%-Bootstrap-Konfidenzintervall, [1]unstandardisierter b-Koeffizient.

Mediation 2: Zusammenhang zwischen Depressivität zu Rehabilitationsbeginn und subjektiver physischer Arbeitsfähigkeit zur 24-Monatskatamnese mit schmerzspezifischer Selbstwirksamkeit (FESS) zur 12-Monatskatamnese als Mediator sowie MPSS und experimentelle Bedingung (Kontrollgruppe vs. Interventionsgruppe) als Kovariaten (*N*=382)

		**Kriterium**						
		***M* (Schmerzspezifische Selbstwirksamkeit)**				***Y* (Subjektive physische Arbeitsfähigkeit)**		
**Prädiktor**		*Koeff.*	*SE*	*p*		*Koeff.*	*SE*	*p*
X (Depressivität)					*c*	-0,28	0,05	<,001
X (Depressivität)	*a*	-0,40	0,04	<,001	*c´*	-0,01	0,04	,772
M (FESS)		-	-	-	*b*	0,66	0,04	<,001
C_1 (MPSS) [1]	f_1	-0,36	0,06	<,001	g_1	-0,14	0,06	,012
C_2 (BED) [1]	f_2	0,18	0,09	,046	g_2	0,01	0,07	,920
		$R_a^2 = 0{,}28$, $F(3, 378) = 47{,}88$, $p < {,}001$				$R_{c'}^2 = 0{,}50$, $F(4, 377) = 92{,}91$, $p < {,}001$		

Vollstandardisierter indirekter Effekt (*ab*)	β	Boot SE	95%-B-KI
	-0,26	0,03	-0,32 - -0,20

Anmerkungen: MPSS = Mainzer Stadienmodell der Schmerzchronifizierung, BED = experimentelle Bedingung; Koeff.: β-Koeffizient, *SE*: Standardfehler, *p*: Signifikanzwert, Boot *SE*: Bootstrap-Standardfehler, 95%-B-KI: 95%-Bootstrap-Konfidenzintervall, [1]unstandardisierter b-Koeffizient.

Mediation 3: Zusammenhang zwischen Depressivität zu Rehabilitationsbeginn und subjektiver psychischer Arbeitsfähigkeit zur 24-Monatskatamnese mit schmerzspezifischer Selbstwirksamkeit (FESS) zur 12-Monatskatamnese als Mediator sowie MPSS und experimentelle Bedingung (Kontrollgruppe vs. Interventionsgruppe) als Kovariaten (*N*=382)

		**Kriterium**						
		***M* (Schmerzspezifische Selbstwirksamkeit)**				***Y* (Subjektive psychische Arbeitsfähigkeit)**		
**Prädiktor**		*Koeff.*	*SE*	*p*		*Koeff.*	*SE*	*p*
X (Depressivität)					*c*	-0,39	0,05	<,001
X (Depressivität)	*a*	-0,40	0,04	<,001	*c´*	-0,24	0,05	<,001
M (FESS)		-	-	-	*b*	0,38	0,05	<,001
C_1 (MPSS) [1]	f_1	-0,36	0,06	<,001	g_1	-0,08	0,07	,253
C_2 (BED) [1]	f_2	0,18	0,09	,046	g_2	0,01	0,09	,997
		$R_a^2 = 0{,}28$, $F(3, 378) = 47{,}88$, $p < {,}001$				$R_{c'}^2 = 0{,}30$, $F(4, 377) = 40{,}92$, $p < {,}001$		

Vollstandardisierter indirekter Effekt (*ab*)	β	Boot SE	95%-B-KI
	-0,15	0,03	-0,21 - -0,10

Anmerkungen: MPSS = Mainzer Stadienmodell der Schmerzchronifizierung, BED = experimentelle Bedingung; Koeff.: β-Koeffizient, *SE*: Standardfehler, *p*: Signifikanzwert, Boot *SE*: Bootstrap-Standardfehler, 95%-B-KI: 95%-Bootstrap-Konfidenzintervall, [1]unstandardisierter b-Koeffizient.
